# Dengue Virus Infections among Haitian and Expatriate Non-governmental Organization Workers — Léogane and Port-au-Prince, Haiti, 2012

**DOI:** 10.1371/journal.pntd.0003269

**Published:** 2014-10-30

**Authors:** Stephanie J. Salyer, Esther M. Ellis, Corvil Salomon, Christophe Bron, Stanley Juin, Ryan R. Hemme, Elizabeth Hunsperger, Emily S. Jentes, Roc Magloire, Kay M. Tomashek, Anne Marie Desormeaux, Jorge L. Muñoz-Jordán, Lesly Etienne, Manuela Beltran, Tyler M. Sharp, Daphne Moffett, Jordan Tappero, Harold S. Margolis, Mark A. Katz

**Affiliations:** 1 Centers for Disease Control and Prevention, Atlanta, Georgia, United States of America; 2 Centers for Disease Control and Prevention, San Juan, Puerto Rico; 3 Ministry of Public Health and Population, Port-au-Prince, Haiti; 4 International Federation of Red Cross and Red Crescent Societies, Port-au-Prince, Haiti; 5 Centers for Disease Control and Prevention, Port-au-Prince, Haiti; 6 Haitian Red Cross, Port-au-Prince, Haiti; U.S. Naval Medical Research Unit No. 2, Indonesia

## Abstract

In October 2012, the Haitian Ministry of Health and the US CDC were notified of 25 recent dengue cases, confirmed by rapid diagnostic tests (RDTs), among non-governmental organization (NGO) workers. We conducted a serosurvey among NGO workers in Léogane and Port-au-Prince to determine the extent of and risk factors for dengue virus infection. Of the total 776 staff from targeted NGOs in Léogane and Port-au-Prince, 173 (22%; 52 expatriates and 121 Haitians) participated. Anti-dengue virus (DENV) IgM antibody was detected in 8 (15%) expatriates and 9 (7%) Haitians, and DENV non-structural protein 1 in one expatriate. Anti-DENV IgG antibody was detected in 162 (94%) participants (79% of expatriates; 100% of Haitians), and confirmed by microneutralization testing as DENV-specific in 17/34 (50%) expatriates and 42/42 (100%) Haitians. Of 254 pupae collected from 68 containers, 65% were *Aedes aegypti*; 27% were *Ae. albopictus*. Few NGO workers reported undertaking mosquito-avoidance action. Our findings underscore the risk of dengue in expatriate workers in Haiti and Haitians themselves.

## Author Summary

Dengue is the most common mosquito-borne viral disease in the world, and caused an estimated 390 million infections and 96 million cases in the tropics and subtropics in 2010. Over the last decade, the number of cases of dengue and the severity of dengue virus infections have increased in the Americas, including the Caribbean, yet little is still known about dengue in Haiti. Following an outbreak of dengue in mostly expatriate NGO workers, the investigators of this study took blood samples from expatriate and Haitian NGO workers living in two cities in Haiti and tested them for evidence of current, recent, and past dengue virus infection. They also investigated the amount and kinds of mosquitoes at homes and work sites. The study found recent infections among some Haitians and expatriates and widespread past infections among all Haitians and most expatriates. It also found that many people were not doing basic things to avoid mosquito bites, like applying mosquito repellent multiple times a day and wearing long sleeves or pants. These findings highlight the likely endemicity of dengue virus in Haiti, and the need to improve knowledge and awareness of dengue prevention among expatriates visiting Haiti and local Haitians.

## Introduction

Dengue is the most common mosquito-borne viral disease in the world, and resulted in an estimated 390 million infections and 96 million symptomatic cases throughout the tropics and subtropics in 2010 [1], [2]. Over the last decade, the incidence and the severity of dengue have increased in the Americas, including the Caribbean [3], [4], where the four dengue virus-types (DENV-1–4) that cause dengue and the mosquitoes (i.e., *Aedes aegypti* and *Ae. albopictus*) that transmit DENV are endemic [1], [5]–[7]. The risk of acquiring dengue can be greatly reduced by following key mosquito avoidance activities, such as applying mosquito repellent multiple times a day and wearing long sleeves, pants or permethrin-treated clothing [8], [9].

Despite an absence of routine systematic surveillance data, dengue is likely endemic in Haiti, as it is in the Dominican Republic, which shares the island of Hispaniola with Haiti. Both *Ae. aegypti* and *Ae. albopictus* have been detected in Haiti, as have all four dengue virus-types [10], [11]. A 2007 study in Port-au-Prince showed that 65% of children <5 years of age had evidence of prior infection with a DENV [12], and a two-year prospective study in an outpatient clinic in Léogane found that 2% of patients presenting with undifferentiated fever tested positive for DENV infection by a rapid diagnostic test (RDT) [13]. Similarly, of 885 patients with acute febrile illness who were admitted to four hospitals in Haiti during 2012–2013, 4% tested positive for DENV infection by RDT [14].

Although dengue has been documented in US military personnel and expatriate relief workers in Haiti in the past two decades [15]–[19], visitors often do not regularly employ mosquito avoidance practices. In a survey conducted among American missionaries returning from Haiti in 2010, only 24% reported using mosquito repellent multiple times a day [15], and in a 1997 study of US military personnel in Haiti only 18% of febrile patients reported always using mosquito repellant [20].

In October 2012, the International Federation of Red Cross and Red Crescent (IFRC) and Red Cross-Haiti alerted the Haitian Ministry of Public Health and Sanitation (French acronym: MSPP) and the US Centers for Disease Control and Prevention (CDC) of 25 recent RDT-positive dengue cases among Haitian and expatriate staff of non-governmental organizations (NGOs) based mostly in Port-au-Prince and Léogane. Seven (28%) of the 25 cases were evacuated from Haiti for advanced medical care. To estimate the incidence of recent and previous DENV infection and identify demographic and behavioral risk factors for infection, we conducted a serologic survey among and administered a questionnaire to Haitian and expatriate NGO workers in Léogane and Port-au-Prince. Additionally, to better understand entomologic risk factors for human infection, we carried out an entomologic investigation around work sites and workers' residences.

## Methods

### Outbreak Investigation

#### Investigation sites

**Figure 1:**
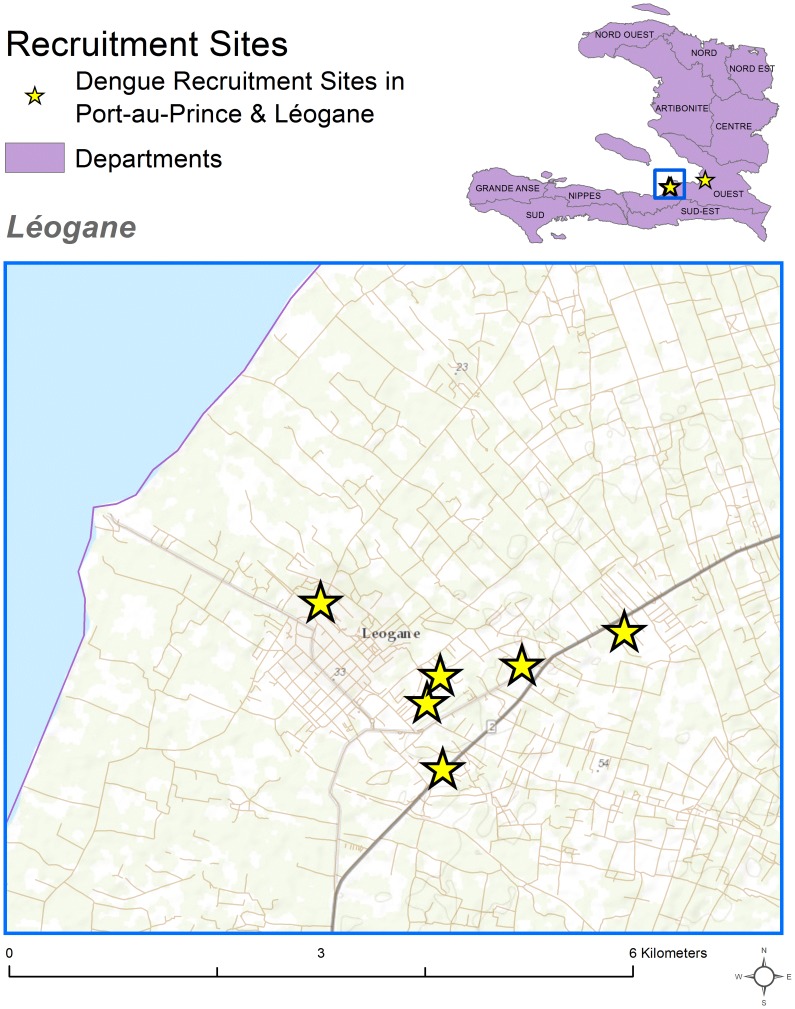
Location of non-governmental organizations that participated in dengue serosurvey, Haiti, 2012.

The investigation was conducted during November 26–December 14, 2012. Because most reported dengue cases were from Léogane, a town approximately 30 kilometers south of Port-au-Prince, we focused our investigation there. All Léogane NGOs were invited and agreed to participate, including IFRC, Red Cross Germany (GRC), Red Cross Swiss (SwRC), Red Cross Spain (SpRC), and Médecins Sans Frontières (MSF). In addition, to increase the number of expatriate participants, we invited IFRC expatriate workers based in Port-au-Prince to participate. In the week prior to the study, each NGO's leadership team explained the importance of the study to its staff, and designated a day when staff could take off from work to participate in the study in one central location. (Figure 1)

#### Questionnaire

For each consenting NGO worker, we administered a standardized questionnaire that collected information on medical history, recent illnesses, knowledge of DENV transmission and mosquito avoidance activities, history of prior vaccination with yellow fever and Japanese encephalitis vaccines, and history of prior dengue, West Nile, or St. Louis encephalitis virus infection. We also asked expatriate workers about dengue educational information they had received prior to arriving in Haiti. The questionnaire was offered in English, French, and Creole, and was either self-administered or read to the participant by investigators. All participants were assigned a unique identifier to link questionnaire responses with laboratory results.

#### Serologic survey

Each participating NGO invited their entire staff (N = 776), including Haitians and expatriates, to participate. We collected a blood specimen for dengue diagnostic testing from all consenting participants, and distributed educational materials on the clinical features of dengue and preventive measures.

#### Laboratory testing

All serum specimens were sent to CDC Dengue Branch for diagnostic testing by CDC DENV-1–4 Real-Time RT-PCR Assay (rRT-PCR) [21], Panbio Dengue Early NS1 antigen capture enzyme-linked immunosorbent assay (ELISA) (Alere Inc.; Waltham, MA) [22], DENV Detect IgM Capture ELISA (InBios International, Inc.; Seattle, WA), and an anti-DENV IgG ELISA [23]. For the IgG ELISA, serum was diluted starting at 1∶40 followed by four-fold dilutions and ending at 1∶655,360. Serum samples with IgG-ELISA titers of 1∶40 or greater were considered to be probable evidence of previous DENV infection. However, due to potential cross-reaction of anti-flavivirus antibodies with DENV antigen [24], we tested a subset of specimens with anti-DENV IgG titers between 1∶160 and 1∶640, but no detectable anti-DENV IgM antibody, by a microneutralization assay [25] to confirm anti-DENV IgG results. We also tested by microneutalization all anti-DENV IgG-positive expatriate specimens with titers ≥1∶640, and a random selection of 40 Haitian anti-DENV IgG-positive specimens with titers >1∶640.

We defined current DENV infection as detection of DENV nucleic acid by rRT-PCR or non-structural protein 1 (NS1) antigen by NS1 ELISA. We defined recent DENV infection as detection of anti-DENV IgM antibody by ELISA, and past DENV infection as detection of anti-DENV IgG antibody by ELISA [2] with subsequent confirmation by microneutralization.

#### Data management and statistical analysis

All data were entered into a Microsoft Access 2010 database (Microsoft Corp, Redmond, WA). All personal identifiers were removed from the database prior to analysis. Dichotomous variables were compared by means of the Fisher's exact tests using IBM SPSS Statistics version 21.0 (Armonk, NY, 2012) to evaluate any risk factors associated with current or recent DENV infection. A P-value<0.05 was considered statistically significant, and any variable with a cell size less than five was rejected from analysis to prevent false findings of statistical significance due to small sample size. Hypergeometric distributions were used to calculate 95% confidence intervals (CIs) around the Haitian specimens that were tested by microneutralization.

#### Entomologic surveillance

We conducted entomologic surveys at all NGO worksites and NGO residences and buildings adjacent to these sites. We also surveyed a convenience sample of Haitian employee residences. At each premise, we counted and characterized the number of water-holding containers by type (e.g., plastic container, water cistern, flower pot). Water-holding containers were classified as being: 1) suitable for immature mosquitoes (both larvae and pupae), but not containing water; 2) containing water but without immature mosquitoes; or 3) containing water and immature mosquitoes. We classified containers by the number of pupae they contained: no pupae, 1–5 pupae, or >5 pupae. Pupae were collected from each container type at each site to identify the mosquito species and the container types that mosquitoes were using for oviposition. Due to logistical constraints, pupae from Haitian employee residences could not be collected. We determined the Premise index – the percentage of premises infested with immature mosquitoes; the Breteau index, defined as the number of containers with immature mosquitoes per 100 premises; and the Container index, defined as the percentage of water-holding containers with immature mosquitoes [26].

#### Human subjects consideration

This investigation was determined to be non-research by both the Haitian National Ethical Review Committee and CDC Human Subjects Advisors; as such, IRB approval was not required. Participants provided either verbal or written consent prior to participation. NGO workers whose houses were inspected for the environmental survey gave written or verbal consent.

Diagnostic test results along with information on how to avoid DENV infection were distributed to participants by email or through an NGO focal point. In addition, we conducted follow-up site visits for all NGOs and NGO staff to share cumulative study findings, implications of the findings, and answer questions about cumulative and individual test results.

## Results

### Demographics and Questionnaire Results

Of 776 NGO workers (106 expatriates and 670 Haitians) in Léogane and Port-au-Prince, 181 (23%) participated in the investigation, including 52 expatriates and 129 Haitians. Of those, 173 (96%) provided a blood specimen for diagnostic testing. The majority of participants were male (76%) and Haitian (71%), and the median age was 33 years (Table 1). Most participants worked in administrative or office duties, construction, or community or field work.

**Table 1:** Demographics, travel history, and vaccination and clinical history of non-governmental organization workers living in Léogane and Port-au-Prince, Haiti, 2012.

	Total		Haitians		Expatriates		p-value*
	n = 181		n = 129		n = 52		
	n	(%)	n	(%)	n	(%)	
**Sex (male)**	137	76%	101	78.3%	36	70%	0.25
**Age, median (range)** †	33 years (19–66)		31 years (19–66)		33 years (24–61)		<0.01
**Occupation** ‡							
Construction	39	22%	31	24%	8	15%	0.23
Community/Field work	23	13%	19	15%	4	8%	0.23
Driver	15	8%	14	11%	1	2%	0.07
Guard	16	9%	15	12%	1	2%	0.04
Hospital/clinic	8	4%	2	2%	6	12%	0.01
Logistics	22	12%	14	11%	8	15%	0.45
Office/admin	53	29%	27	21%	26	50%	<0.01
Water and Sanitation	22	12%	11	9%	11	21%	0.02
Other	24	13%	16	12%	8	15%	0.63
**Born in a country with known dengue risk**	140	77%	128	99%	11	21%	<0.01
**Ever lived in or traveled to a country with known dengue risk**	79	44%	30	23%	49	94%	<0.01
**Vaccine History**							
Yellow fever	51	28%	5	4%	46	89%	<0.01
Japanese encephalitis	12	7%	0	0%	12	23%	<0.01
**Ill in last 90 days**	40	22%	18	14%	22	42%	<0.01
with fever	21	12%	8	6%	13	25%	0.01
**Previous diagnosis of dengue** §	10	6%	0	0%	10	19%	<0.01

* p-value was calculated using a 2-tailed Fisher's exact test; the p-value for age and work site was calculated using Chi square with Yate's correction.

† 10 Haitians and 2 expatriates did not report their age.

‡ 5 Haitians and 1 expatriate did not report their occupation.

§ includes people who knew about their recent diagnosis (within the last 90 days).

Less than a quarter (21%) of expatriates reported being born in a dengue-endemic country. Nearly all expatriates (94%) but less than a quarter (23%) of Haitians reported ever living in (>1 month) or traveling to (>1 week) a dengue-endemic country other than Haiti in their lifetime. Nearly all expatriates (96%), but less than half (39%) of Haitians, reported ever hearing of dengue. Expatriates reported greater knowledge of DENV transmission (89% vs. 29%) and dengue prevention (96% vs. 13%) compared with Haitians. While 6% of expatriates reported a previous dengue diagnosis, no Haitians reported ever being diagnosed with dengue. Overall, 89% and 23% of expatriate staff reported receiving a yellow fever or Japanese encephalitis vaccination, respectively. In contrast, among Haitian staff, only 4% and 0% reported receiving a yellow fever vaccination or Japanese encephalitis vaccination, respectively.

The majority (87%) of expatriates and half (47%) of Haitians reported using mosquito repellent, but less than half (44%) of expatriates and only a small proportion (9%) of Haitians reported using mosquito repellent multiple times a day (Table 2). While most expatriates and Haitians reported using a bed net, only a small percentage of expatriates and Haitians (10% and 2%, respectively) reported using permethrin-treated clothing. Of the 52 expatriate workers, most (87%) said they had made a travel consultation prior to their current trip to Haiti. Of the 45 workers who reported making a travel consultation, approximately half (47%) went to a travel medicine clinic, 71% received mosquito-avoidance information during their consultation, and 39% received information about dengue.

**Table 2:** Dengue knowledge, mosquito avoidance strategies and personal protective measures of non-governmental organization workers living in Léogane and Port-au-Prince, Haiti, 2012.

	Total		Haitians		Expatriates		p-value*
	n = 181		n = 129		n = 52		
	n	%†	n	%†	n	%†	
**Dengue knowledge**							
Ever heard of dengue	100	55%	50	39%	50	96%	<0.01
Knowledge of dengue transmission	83	46%	37	29%	46	89%	<0.01
Knowledge of dengue prevention	67	37%	17	13%	50	96%	<0.01
**Mosquito Avoidance Strategy**							
Wearing long sleeves	57	32%	35	27%	22	42%	0.05
Wearing long pants	68	38%	36	28%	32	62%	<0.01
Mosquito repellent	106	59%	61	47%	45	87%	<0.01
*multiple times a day*	34	19%	11	9%	23	44%	<0.01
Bed nets	118	65%	90	70%	28	54%	0.06
Aerosol sprays	58	32%	34	26%	24	46%	0.01
Treated Clothing	7	4%	2	2%	5	10%	0.02
None	14	8%	12	9%	2	4%	0.36
**Presence/use of the following**							
Screens at							
*Hang out place*	16	9%	10	8%	6	12%	0.40
*Sleep place*	74	41%	37	29%	37	71%	<0.01
*Work site*	67	37%	47	36%	20	39%	0.87
Air conditioning at							
*Hang out place*	15	8%	6	5%	9	17%	0.01
*Sleep place*	33	18%	5	4%	28	54%	<0.01
*Work site*	56	31%	26	20%	30	58%	<0.01
Open water source nearby							
*Hang out place*	47	26%	28	22%	19	37%	0.60
*Sleep place*	65	36%	45	35%	20	39%	0.73
*Work site*	49	27%	32	25%	17	33%	0.36
Standing water source nearby							
*Hang out place*	45	25%	22	17%	23	44%	<0.01
*Sleep place*	55	30%	29	23%	26	50%	<0.01
*Work site*	57	32%	30	23%	27	52%	<0.01
Tires or trash nearby							
*Hang out place*	38	21%	19	15%	19	37%	0.002
*Sleep place*	41	23%	22	17%	19	37%	0.006
*Work site*	43	24%	22	17%	21	40%	0.002

*p-value was calculated using a 2-tailed Fisher's exact test.

† all percentages were rounded to the nearest whole number.

Compared with their Haitian colleagues, more expatriates reported having screens on their windows or doors, and air conditioning at their sleep site. Expatriates also reported more standing water and trash near their work site, off-hours ‘hang-out’ places, and sleep site as compared to Haitians (Table 2).

### Serologic Survey

DENV nucleic acid was not detected in any of the 173 NGO workers who provided blood specimens for dengue diagnostic testing. Both NS1 and anti-DENV IgM antibody were detected in one asymptomatic expatriate. Anti-DENV IgM antibody was detected in 17 (10%) NGO workers (8 [15%] expatriates and 9 [7%] Haitians) (Table 3). Of the 17 participants with evidence of current and/or recent DENV infection, six (35%) participants (five expatriates and one Haitian) reported being ill in the past 90 days, five (29%) reported missing at least one day of work, and three (18%) were hospitalized and subsequently required medical evacuation to the Dominican Republic. Of these three evacuated participants, two had dengue with warning signs: one had menorrhagia and the other had a pleural effusion.

**Table 3:** Dengue diagnostic test results of non-governmental organization workers living in Léogane and Port-au-Prince, Haiti, 2012.*

	Total		Haitians		Expatriates		p-value†
	n = 173		n = 121		n = 52		
	n	%	n	%	n	%	
DENV PCR positive‡	0	0%	0	0%	0	0%	-
NS1 ELISA positive§	1	1%	0	0%	1	2%	0.30
IgM ELISA positive¶	17	10%	9	7%	8	15%	0.16
IgG ELISA positive#	161	93%	121	100%	41	79%	<0.01

*DENV denotes dengue virus. NS1 denotes the DENV non-structural protein 1. ELISA denotes an enzyme-linked immunosorbent assay.

† p-value was calculated using a 2-tailed Fisher's exact test.

‡ Positive by DENV-1–4 Real-Time rt-PCR.

§ Positive by the NS1antigen capture ELISA.

¶ Positive by the DENV Detect immunoglobulin M Capture ELISA.

# Positive by an anti-dengue virus immunoglobulin G ELISA. All expatriate and 40 Haitian samples that were IgG ELISA positive received confirmatory testing by microneutralization.

Of 173 specimens tested, 161 (93%) had detectable anti-DENV IgG antibody, including 41 (79%) expatriates and all 121 Haitians. Prior DENV infection was confirmed by microneutralization assay in 17 (50%) of the 34 IgG-positive/IgM-negative specimens from expatriates, and in all 42 randomly selected specimens from the 121 IgG-positive Haitians (95% confidence interval [CI]: 94.5%–100%) (Table 4).

**Table 4:** Confirmation of anti-DENV IgG positive ELISA results, Haiti, 2012.

IgG Positive Cases	Haitians*		Expatriates		Total	
	n = 121		n = 41		n = 162	
	n	%	n	%	n	%
**With recent infection (anti-DENV IgM positive)†**	**9**	**7.4%**	**7**	**17.1%**	**16**	**9.9%**
**No recent infection, tested by microneutralization**	**42**	**34.7%**	**34**	**82.9%**	**76**	**46.9%**
**No recent infection, not tested by microneutralization**	**70**	**57.9%**	**0**	**0.0%**	**70**	**43.2%**
Micronetralization results**	**n = 42**		**n = 41**		**n = 59**	
Positive	42	100.0%	17	50.0%	59	77.6%
*DENV cross reactive*	30	71.4%	11	32.4%	41	53.9%
*DENV-1*	1	2.4%	1	2.9%	2	2.6%
*Primary DENV-1*	0	0.0%	1	2.9%	1	1.3%
*DENV-2*	8	19.0%	3	8.8%	11	14.5%
*DENV-3*	3	7.1%	0	0.0%	3	3.9%
*DENV-4*	0	0.0%	0	0.0%	0	0.0%
*Primary DENV-4*	0	0.0%	1	2.9%	1	1.3%
Negative	0	0.0%	17	50.0%	17	22.4%

*42 specimens were randomly selected from the 121 IgG-positive Haitians (95% confidence interval [CI]: 94.5%–100%).

† Samples that were anti-IGM ELISA positive were not tested by microneutralization since they were considered recent DENV infections.

** DENV cross reactive = positive neutralization titers to more than one DENV serotype without a four-fold difference between DENV serotypes to determine the predominant serotype; Primary DENV = reactivity to only one DENV serotype.

### Risk Analysis

Participants who reported working near “open water sources” had greater odds of having had a current and/or recent DENV infection (odds ratio [OR] = 3.6, 95% CI = 1.3–10.1; Table 3). Participants who reported using mosquito repellent multiple times a day (OR = 3.5, 95% CI = 1.22–10.04), having very good knowledge of infectious disease in Haiti (OR = 3.6, 95% CI 1.16–10.98), and knowing how to prevent mosquito bites (OR = 6.2, 95% CI 1.92–19.72) had greater odds of having had a current and/or recent DENV infection. No other risk factors were found to be statistically significant (Table 5).

**Table 5:** Risk factors for current and/or recent dengue virus (DENV) infection in non-governmental organization (NGO) workers living in Léogane and Port-au-Prince, Haiti, 2012.

Variable	NGO workers with recent infection†		NGO workers without recent infection		Crude OR* (95% CI)‡	p-value
	n = 17		n = 156		n = 181	
	n	%	n	%		
**Sex (male)**	11	65%	120	77%	0.6 (0.19–1.59)	0.37
**Expatriates**	8	47%	44	28%	2.3 (0.82–6.24)	0.16
**Occupation**						
Indoor setting (ex. Office/admin)	1	6%	32	21%	0.2 (0.03–1.9)	0.20
Outdoor setting (ex. Construction)	8	47%	68	44%	1.2 (0.43–3.38)	0.80
Mixed setting (both indoor and outdoor)	7	41%	50	32%	1.6 (0.55–4.42)	0.42
**Vaccination History** (YF or JPE)	7	41%	43	28%	1.8 (0.66–5.14)	0.27
**Previously lived in or traveled to Other Dengue Endemic Regions**	9	53%	70	45%	1.4 (0.51–3.8)	0.61
**Lived in or Travel to Dengue Endemic Regions** (incl. Haiti)	15	88%	155	99%	0.48 (0.004–0.57)	0.03
**Environmental factors at work place**						
Screens on doors/windows	8	47%	59	38%	1.5 (0.53–4.00)	0.60
Air-conditioning	6	35%	47	30%	1.3 (0.44–3.62)	0.78
Open water source nearby	9	53%	37	24%	3.6 (1.30–10.05)	0.02
Standing water source nearby	7	41%	45	29%	1.7 (0.62–4.82)	0.30
**Reported knowledge of**						
Infectious disease in Haiti (very good)	6	35%	23	15%	3.6 (1.16–10.98)	0.03
Mosquito bite prevention	9	53%	31	20%	6.2 (1.92–19.72)	0.002
**Mosquito avoidance strategies employed**						
Long sleeves	5	29%	50	32%	0.9 (0.30–2.64)	1.00
Long pants	5	29%	61	39%	0.7 (0.22–1.93)	0.60
Bed net	11	65%	101	65%	1.0 (0.35–2.85)	1.00
Mosquito repellent use						
*multiple times a day*	7	41%	26	17%	3.5 (1.22–10.04)	0.02

*OR, odds ratio.

† Recent infection is any participant with a positive anti-DENV IgM or non-structural protein 1 (NS1) result.

‡ Significance level, p<0.05. Univariate analysis using Fisher's exact test was used to assess risk factors for recent infection, and 95% confidence intervals (CI) were based on the modeling accounted for the sampling design. Only significant variables with a cell size of 5 or greater were retained.

### Entomology Survey

One hundred premises were surveyed, including 8 NGO work sites and 28 adjacent buildings, 8 NGO residences and 27 adjacent buildings, and 29 Haitian employee residences. In total, 2,664 containers were inspected for immature mosquitoes. Of these containers, 756 (28%) contained water, of which 198 (26%) contained immature mosquitoes. We collected 254 pupae from 68 water-holding containers; *Ae. aegypti* was the most abundant mosquito species identified (65%), followed by *Ae. albopictus* (27%). The remaining 8% of mosquitoes identified were *Ae. mediovittatus*, *Culex* species, or were unidentifiable. Vector indices were similar between NGO work sites and residences. All mosquito abundance indices were elevated. For all premises combined, the Premise index was 61%, the Container index was 26%, and the Breteau index was 198 (Table 6). Pupae were found in 46% of tires, 29% of cans, 28% of water drums, 21% of cisterns, and 20% plastic containers that held water.

**Table 6:** Results from the entomological survey conducted in Léogane, Haiti, December 5–13, 2012.

Indicator	NGO residences	NGO work sites and offices	Individual* Haitian employee residences	Total Combined
**Premise Index**	69%	56%	59%	**61%**
Premises visited	35	36	29	**100**
Premises positive for larvae/pupae	24	20	17	**61**
**Container Index**	33%	27%	16%	**26%**
Containers surveyed	830	1,202	632	**2,664**
No. of containers holding water	230	338	188	**756**
No. of containers with larvae/pupae	77	91	30	**198**
**Breteau Index**	220	253	103	**198**

*Entomologic surveys conducted at all NGO worksites and NGO residences included surveys of adjacent buildings. Adjacent buildings were not surveyed for Haitian employee residences.

Premise index = the percentage of premises infested with immature mosquitoes.

Container index = the percentage of water-holding containers with immature mosquitoes.

Breteau index = the number of containers with immature mosquitoes per 100 premises.

## Discussion

In our investigation of NGO workers in Léogane and Port-au-Prince, Haiti, we found that a substantial proportion (15% of expatriates and 7% of Haitians) had recently been infected with a DENV. Six of the infected workers reported being ill, and three required evacuation from Haiti for medical care. This rate of recent DENV infection is similar to findings from two previous studies in Haiti that reported rates of infection as high as 25% in expatriates and 29% in military personnel (12,15). These findings demonstrate the risk of dengue for visitors to and residents of Haiti, and also illustrate the potential economic consequences of dengue through missed work days, hospitalization, and medical evacuation [27], [28].

While there is no vaccine to prevent dengue, people at risk for DENV infection, such as the NGO workers in our investigation, can reduce their chance of getting infected through a number of preventive measures like applying mosquito repellent multiple times a day and wearing permethrin-treated clothing [8], [9]. The NGO workers in our investigation variably employed these preventive measures: less than half of expatriates reported using mosquito repellent multiple times a day, and only 10% of expatriates used permethrin-treated clothing. The majority of expatriates in our investigation had a pre-travel health consultation. This rate is higher than previous reports of pre-travel health consultations among US citizens who traveled to countries with elevated public health risks [29], [30]. However, in our investigation, less than half of expatriates received information about dengue during their pre-travel consultation. Improving pre-deployment education of expatriate NGO workers could increase the likelihood that they will employ preventive measures once they are in the field.

All Haitian NGO workers had evidence of prior DENV infection, providing further evidence of dengue endemicity in Haiti. While our investigation and previous studies [11], [12], [14], [31] collectively provide strong evidence for dengue endemicity in Haiti, questions remain about the clinical course of dengue among Haitians. Some studies have hypothesized that Haitians and persons of African descent are less likely to experience severe dengue [11], [32]. In fact, in our investigation, some Haitian NGO staff said that dengue was not a health threat to Haitians and therefore declined to participate. In our investigation, only one Haitian (11%) with a recent DENV infection reported dengue-like symptoms, and no Haitians reported symptoms of severe dengue. However, hospital and clinic-based surveillance conducted over the last two years in Haiti has shown that dengue is associated with both clinic visits and hospital admissions among Haitians [13], [14]. Broader surveillance should be undertaken in Haiti to better understand the burden and clinical course of dengue in Haitians.

Among all the sites we inspected in Léogane, the Premise and Breteau indices were 61% and 198, respectively, reflecting an increased risk for DENV transmission [2], [33]. These findings, according to WHO guidelines, indicate a need to prioritize vector control [26]. The density of immature vectors found in Léogane was greater than what was reported in a survey conducted in Port-au-Prince in May 2011 [34]. In our risk factor analysis, we found that NGO staff who worked near open water had an increased risk of DENV infection. Although this question did not clearly define open water with examples, anecdotally respondents interpreted this question to mean open containers filled with water. Other studies, including a recent study conducted in Saudi Arabia [35], have identified proximity to standing water as a risk factor for DENV infection. Efforts should be made by NGOs and individuals to eliminate mosquito-breeding habitats by systematically reducing standing water in containers around worksites and residences. While vector control has had mixed results with regard to decreasing DENV infections [36], it is still effective at reducing DENV-transmitting mosquitoes by eliminating container habitats [37], [38]. However, dengue risk perceptions need to be addressed within these communities to make these efforts sustainable [39], [40].

We found some unexpected results in our risk factor analysis. The use of mosquito repellent multiple times a day was associated with DENV infection. This finding is most likely due to sampling bias; four (24%) of the recently infected participants in this investigation had received a diagnosis of dengue in the three months prior to our investigation and subsequently received education about dengue. Their responses to these questions likely reflected a change in risk perceptions and an awareness of dengue that they acquired after receiving their diagnosis [41], [42]. Because the questionnaire did not distinguish whether information acquired after receiving a diagnosis of dengue had an effect on knowledge or practice, our findings related to this risk factor are likely spurious. Avoidance of mosquito bites by use of mosquito repellent is a widely supported measure for dengue prevention [8], [9], [43].

Our investigation was subject to several limitations. Because we used a convenience sample and less than half of all NGO workers at the Léogane-based NGOs and IFRC in Port-au-Prince participated, our results may not be representative of all workers at these NGOs. Also, we were not able to systematically evaluate whether there were demographic differences between participants and non-participants. While anecdotally some NGO workers declined to participate because of doubts about the relevance of dengue in Haiti, others were unavailable at the time of the survey. In addition, the investigation was conducted only among NGO workers in the two cities, and therefore our results may be not generalizable to other parts of the expatriate and Haitian population. Our analysis of risk factors for DENV infection was limited by a relatively small sample size. Finally, we were not able to link the results from the serosurvey with our findings from the entomologic investigation.

Our findings underscore the risk of dengue in expatriate workers in Haiti. Expatriate NGO staff should be briefed on dengue risk and prevention measures prior to their arrival in Haiti, and NGOs should systematically employ vector control measures at their work sites and residences to reduce mosquito populations. We found evidence of acute dengue virus infections in Haitians and we found a high rate of previous infection among Haitians. Surveillance and research should be undertaken to better understand clinical dengue in Haitians.

## Supporting Information

Checklist S1 STROBE checklist.
(DOC)
